# Corifollitropin alpha, clomiphene citrate and dydrogesterone without daily gonadotrophin: a new option of a friendly protocol for high-responder oocyte donors

**DOI:** 10.5935/1518-0557.20210082

**Published:** 2022

**Authors:** Anderson Sanches de Melo, Camilla Teles Vidal de Paula, Thiago Lopes dos Santos, Victor Antonio Costa Faria, Marcelo Augusto Feres Rufato, Rebecca Pontelo Barboza, Jorge Barreto

**Affiliations:** 1 CEFERP - Fertility Center of Ribeirão Preto. Ribeirão Preto, São Paulo, Brazil

**Keywords:** oocyte donors, in vitro fertilization, progestin-primed ovarian stimulation, ovarian hyperstimulation syndrome, corifollitropin alpha

## Abstract

**Objective:**

To compare the number of oocytes obtained in the follicular puncture of high- responder oocyte donors, submitted to ovarian stimulation for in vitro fertilization (IVF) in two different protocols: Friendly and Conventional.

**Methods:**

There were one hundred-and-eight infertile egg-donor women, aged between 21 and 35 years, undergoing IVF in this retrospective cohort study. The women were divided into two groups: 1) Friendly protocol: controlled ovarian stimulation (COS) with corifollitropin alpha, clomiphene citrate and dydrogesterone without daily rFSH (n=52) and 2) In the Conventional protocol, we had COS with menotropin daily and ganirelix (n=66). We assessed age, body mass index, time and cause of infertility, antral follicle count (AFC) by three-dimensional ultrasound, number of visits to the clinic, COS duration, number of follicles ≥14mm on the trigger day, early ovulation frequency, number of mature oocytes, number of cryopreserved embryos, clinical pregnancy rate, frequency of OHSS.

**Results:**

The ovulatory factor was higher in women in the Conventional protocol (*p*=0.03), and the tubal factor (*p*=0.02) was higher in the Friendly protocol group. The number of visits to the clinic was lower among women in the Friendly protocol (*p*=0.04). The number of mature eggs, the clinical pregnancy rate and the frequency of OHSS were similar between the groups. The number of frozen embryos was higher in the Friendly group (p=0.02). The regression model demonstrated that the ovulatory factor, the tubal factor and the number of visits to the clinic were not predictors of the number of mature oocytes. Only AFC was an independent predictor of the number of meiosis II oocytes (*p*<0.01).

**Conclusions:**

The Friendly protocol seems to be as safe and effective as the Conventional protocol for infertile high-responder oocyte donors, resulting in a similar number of mature oocytes and OHSS incidence.

## INTRODUCTION

Conventional controlled ovarian stimulation (COS) for oocyte donation includes the use of gonadotropins for multifollicular growth, associated with the use of a gonadotropin-releasing hormone (GnRH) agonist (often the antagonist) to prevent early ovulation. As these drugs are for injectable use, and due to the frequent need for ultrasound assessment in human reproduction clinics (Massin, 2017), conventional COS can discourage the development of oocyte donation programs, especially in the COVID-19 era, when distancing and social isolation measures are needed (Lewnard & Lo, 2020).

To encourage the development of oocyte donation programs, it is important to use protocols that reduce the number of injections, the number of visits to the clinic, the risk of ovarian hyperstimulation syndrome (OHSS), the financial cost and fear of infection by COVID-19. In this context, the use of long-term gonadotropin (corifollitropin alpha) can contribute to a reduction in the number of injections. When corifollitropin alpha (Zander-Fox *et al*., 2018) or recombinant FSH (Bechtejew *et al*., 2017) are used in combination with oral clomiphene citrate, a reduction in the total daily dose of gonadotropins to complement COS and decrease the risk of ovarian hyperstimulation syndrome (OHSS). These effects can be attractive to potential oocyte donors who generally have increased antral follicle count (AFC) and greater risk for OHSS (Practice Committee of the American Society for Reproductive Medicine, 2016).

On the other hand, corifollitropin alpha is contraindicated in women with AFC> 20 (hyper-responders) due to the risk of OHSS and, regardless of the protocol used, oocyte donors are at increased risk for OHSS. However, with the freeze-all strategy and the trigger with GnRH agonist there was an important reduction in OHSS-related complications (Davenport *et al*., 2017). In addition, studies that evaluated the use of corifollitropin alpha in hyper responders have included the use of daily gonadotropin to complete COS (Hwang *et al*., 2018; Revelli *et al*., 2020) a characteristic that may contribute to a higher risk of OHSS.

The association of corifollitropin alpha with clomiphene citrate can reduce the number of injections, but the subcutaneous applications are still necessary due to the daily use of the GnRH antagonist. An easy, comfortable, inexpensive and practical alternative to pituitary block during COS is the oral use of progestogens (Yu *et al*., 2018). Both the use of micronized progesterone and medroxyprogesterone acetate have been shown to be effective during COS. However, the use of micronized progesterone can cause a cross-reaction in the measurement of serum progesterone, making it difficult to detect early luteinization (Zhu *et al*., 2015). The medroxyprogesterone acetate is associated with more intense pituitary suppression, which requires a higher dose of gonadotropins and can also increase the duration of COS. For this reason, it is important to evaluate other progestogens for pituitary block (Kuang *et al*., 2015).

Dydrogesterone is an inexpensive natural progesterone and does not have the effects previously mentioned for other progestogens. Although 10-20mg/day of dydrogesterone does not inhibit ovulation, its use as an alternative for pituitary block in COS has been shown to be effective and safe in women with normal ovarian reserve (Yu *et al*., 2018). In addition to reducing the number of injections, the use of progestogens could contribute reducing the number of returns to the clinic when associated with corifollitropin alpha and clomiphene citrate, since pituitary block is performed since the beginning of COS and the dose of gonadotropin/clomiphene citrate are fixed (Massin, 2017).

In parallel, a new scenario presents itself in the daily life of humanity: the COVID-19 pandemic. After controlling this infection, many people will change their behavior, avoiding crowded locations and reducing non-essential commuting (Lewnard & Lo, 2020). Thus, protocols that reduce the number of visits to the clinic during COS and provide greater comfort without reducing effectiveness can contribute to the expansion of oocyte donation programs. Despite this, there are no studies that evaluated the simultaneous use of corifollitropin alpha with clomiphene citrate and dydrogesterone associated with the GnRH agonist trigger and freeze all in oocyte donors. Thus, this study aims to compare the number of oocytes obtained in the follicular puncture of high responder oocyte donors submitted to controlled ovarian stimulation (COS) for IVF in two different protocols: Friendly (corifollitropin alpha + clomiphene citrate + dydrogesterone without daily gonadotropin) and Conventional (daily recombinant FSH + GnRH antagonist). In addition, we aim to evaluate the frequency of ovarian hyperstimulation syndrome (OHSS), the number of visits to the clinic and the clinical pregnancy rates.

## MATERIALS AND METHODS

### Study design and context

This is a retrospective cohort study conducted with infertile oocyte donors submitted to IVF from January/2018 to December/2019. We obtained the data through the review of medical records, and the study was approved by the local ethics committee.

In our country, local ethics regulations for donors do not authorize a commercial relationship (there is no financial compensation for donors). Thus, all egg-donor women included in this study had infertility, and for them IVF was indicated as treatment. In these cases, the woman donated half of her eggs and, in return, the recipient paid part of her treatment, anonymously. In the situation of having an odd number of eggs, the donor received one egg more than the number donated.

### Participants

Infertile egg donor women aged 21 to 35 years, submitted to IVF due to polycystic ovary syndrome or unexplained infertility or/and bilateral tubal obstruction or/and male factor were eligible for the present study. Exclusion criteria were antral follicle count (AFC) by transvaginal ultrasound <20 follicles, previous ovarian stimulation for IVF, metabolic disorders related to polycystic ovary syndrome, absence of one ovary, severe male factor (mobile sperm count <1 million or need for surgical sperm acquisition), known hereditary or genetic diseases, infectious diseases (hepatitis B or C, HTLV, HIV, VDRL), infertility time longer than 5 years, smoking, previous use of hormonal contraceptives in the last three months, known endometrial diseases (polyp, fibroids), chronic diseases, adenomyosis and/or endometriosis.

### Protocols

The women included were divided into two groups: 1) Friendly Protocol: COS with corifollitropin alpha, clomiphene citrate and dydrogesterone without daily gonadotropin; 2) Conventional Protocol: COS with menotropin and ganirelix.

All women included were evaluated with transvaginal ultrasound for AFC until the fourth day of the menstrual cycle. In the absence of follicles ≥10mm, the woman started one of the COS regimens, as described below. Half of the mature eggs (meiosis II) obtained were donated anonymously and the other half was for their own use; in cases of an odd number, the donor received half + one of the mature eggs.

### Friendly Protocol

COS included a single subcutaneous dose of 100µg corifollitropin alpha (body weight ≤60 kg or antral follicle count >30) or 150µg (Elonva^®^, Shering-Plough, Ravensburg, Germany) (body weight >60kg and follicle count between 20 and 30) associated with daily use of clomiphene citrate (Indux^®^, EMS. Hortolândia, Brazil) 100 mg/day and dydrogesterone 20 mg/day (Duphaston^®^. Abott, Chicago, USA) since the beginning of COS. On the eighth day after using the injectable gonadotropin, an ultrasound scan was performed to assess follicular growth: if the largest follicle was <14mm, 75-150IU/day, menotropin (Merional^®^, IBSA Insitute Biochimique, Lamone, Switzerland) was used until obtaining two or more follicles with an average diameter of 18mm. At this point, the trigger was performed with 02 ampoules of triptorelin (Gonapeptyl^®^, Ferring GmbH, Kiel, Germany) and the egg collection was performed after 34-36 hours. After 2-4 hours of ovarian puncture, ICSI was performed, and the embryos were vitrified on the third day (D3) or in the blastocyst stage.

### Conventional Protocol

The women received an initial dose of 300IU menotropin (Merional^®^) for four days, and on the fifth day of induction, the dose was reduced to 150IU/day. The first ultrasound to assess follicular development was performed between the fifth and the sixth day of induction. After identifying 02 follicles ≥14mm or 01 ≥16mm, 01 ampoule/day of ganirelix (Orgalutran^®^, Merck Sharp & Dohme B.V., Netherlands) was started up to 24 hours before the trigger. In the presence of two or more follicles with an average diameter of 18mm, a trigger with triptorelin (Gonapeptyl^®^, Ferring GmbH, Kiel, Germany) was prescribed and the egg collection was performed after 34-36 hours. After 2-4 hours of ovarian puncture, ICSI was performed, and the embryos were vitrified on the third day (D3) or on the blastocyst stage.

### Embryo transfer

Between the first and fourth day of the cycle following the egg retrieve (or up to three cycles after ovarian puncture), all participants were instructed to start estradiol hemihydrate (0.6mg/g) (4.5mg/day = 6 puffs twice day) (Oestrogel^®^ - Besins Manufacturing - Drogenbos - Belgium) for 8 to 10 days, at which point a transvaginal ultrasound was performed. When the endometrium was trilaminar and with a thickness ≥7mm, we started vaginal micronized progesterone 600mg/day (Utrogestan^®^ - Besins Manufacturing - Drogenbos - Belgium). Embryo transfer occured after three or four days (for embryos frozen on D3) and five or six days (for frozen blastocysts) after starting micronized progesterone, and the number of embryos was decided by the patient/couple (maximum of two embryos/transfers). After 14 days, we ran a pregnancy test to confirm the biochemical pregnancy. If a pregnancy test was positive, we performed a transvaginal ultrasound after two to four weeks to confirm clinical pregnancy.

### Clinical and laboratory variables

All the women in the sample were submitted to evaluation of age, body mass index (BMI) [Weight (kg)/(height - m)^2^], duration and cause of infertility (ovulatory factor was considered women with polycystic ovary syndrome) and AFC by three-dimensional ultrasound using the Voluson S8 (GE) equipment.

Other variables analyzed were the number of visits to the clinic, duration of COS, number of follicles ≥14 mm on the day of the trigger, frequency of early ovulation (observation of a suggestive image of the corpus luteum on the day of oocyte uptake), number of mature oocytes (meiosis II), number of cryopreserved embryos, clinical pregnancy rate (presence of gestational sac with embryonic heart activity, after four weeks of embryo transfer), frequency of women with OHSS [mild: presence of pain (relieves with scopolamine and/or paracetamol) and/or nausea and/or vomiting; moderate: increase in abdominal volume with ultrasonographic evidence of ascites, pain (relieves with codeine), without the need for hospitalization; severe: need for hospitalization due to respiratory distress and/or oliguria, hemoconcentration) (Golan *et al*., 1989).

### Bias

In order to avoid a selection bias, we considered all oocyte donors submitted to IVF during the period covered in the present study. For women submitted to more than one cycle, only the information from the first IVF was considered for analysis.

### Sample calculation

Considering that 50-70% of ovarian follicles respond to exogenous gonadotropin stimulation (Salih Joelsson *et al*., 2019), it is expected that each participant with ≥20 antral follicles will provide at least 10 to 14 eggs per oocyte retrieval. As there are no comparative trials of the two protocols used in this study, we hypothesized that women submitted to the conventional protocol provide an average of 12 mature oocytes. Thus, in order to detect a difference of 02 oocytes between groups, with α 5% and power 80%, 49 patients would be needed per group (total: 108).

### Statistical analysis

To check the normal distribution of the samples, we used the Kolmogorov-Smirnov and the Shapiro-Wilk test. For variables with normal distribution, we used the unpaired t-test for comparison; in the absence of a normal distribution, we used the Mann-Whitney test. The quantitative variables were represented as mean and standard deviation. For qualitative variables, we used the Fisher and Chi-square tests. The level of significance adopted was 5% with a power of 80%. In addition, we ran a multivariate analysis to assess the prediction of the covariables: age, time and cause of infertility and the number of visits to the clinic on the number of mature oocytes. We ran the statistical analysis with the aid of the SPSS 16.0 for Windows (SPSS Inc., Chicago, Illinois, USA), SAS 9.0 (SAS Institue Inc., North Caroline University, USA) and the GraphPad 5.0 for Windows (GraphPad Software, San Diego, California, USA).

## RESULTS

### Study flow chart

**Figure 1:**
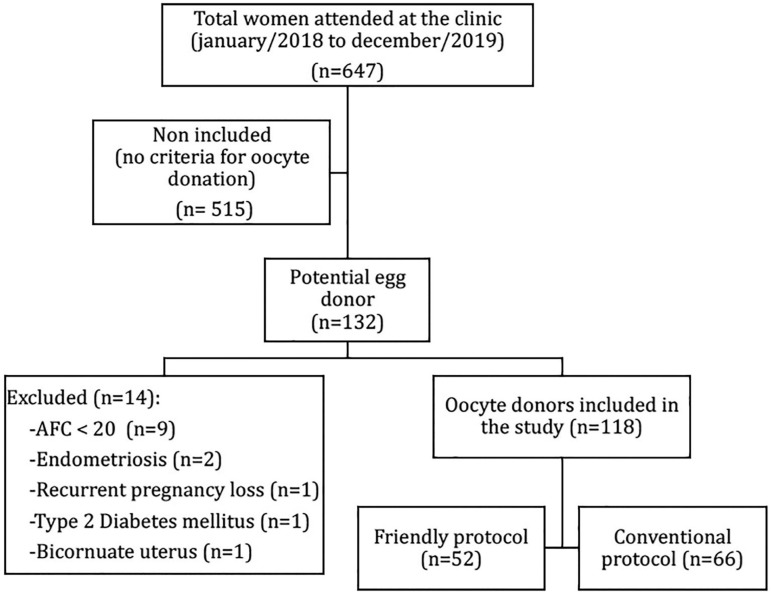
Study flow chart. (AFC: antral follicle count)

Among the women evaluated during the study period (n=647), one hundred and thirty-two met the criteria for oocyte donation. Of these, 14 were excluded (09 had AFC <20 at baseline evaluation; 02 had endometriosis; 01 had recurrent pregnancy loss; 01 had type-2 diabetes mellitus; 01 had bicornuate uterus) and 118 were divided into two groups according to the COS protocol: Friendly (n=52) or Conventional (n=66) (Figure 1).

### Clinical, laboratory and ultrasound variables

Among the causes of infertility, the frequency of ovulatory factor was higher in women in the Conventional protocol (Friendly protocol: 32.5% *vs*. Conventional protocol: 62.5%, *p*=0.03) and the presence of the tubal factor (Friendly protocol: 63.6% *vs*. Conventional protocol: 36.4%, *p*=0.02) was higher in the Friendly protocol group. The number of visits to the clinic was lower among women from the Friendly protocol (Friendly protocol: 2.9±0.5 *vs*. Conventional protocol: 5.3±2.3, *p*=0.04) (Table 1). Age and BMI were similar between the groups.

**Table 1.** Clinical, ultrasound and laboratory variables of two ovarian stimulation protocols in an oocyte donation program.

Variable	Friendly Protocol (n=52)	Conventional Protocol (n=66)	*p*
**Age (years)**	30±3.8	29.1±4.9	NS
**Time of infertility (years)**	4.4±3.4	4.5±3.2	NS
**Cause of infertility**			
**Male (%)**	49.2	50.8	NS
**Ovulatory (%)**	37.5	62.5	0.03
**Tubal (%)**	63.6	36.4	0.02
**Other (%)**	2.8	3.1	NS
**BMI (Kg/m^2^)**	26.3±4.2	26.9±4.9	NS
**Days of induction**	10.4±1.1	10.5±1.2	NS
**Number of visits to the clinic**	2.9±0.5	5.3±2.3	0.04
**Follicles ≥ 14 mm (hCG day)**	14.7±5.5	12.7±6.2	NS
**Early ovulation**	1.9	1.5	NS
**Number of MII oocytes**	18.5±10.1	17.2±9.2	NS
**Number of frozen embryos**	7±4,7	5,3±2,2	0.02
**Clinical pregnancy rate [n(%)]**	20 (40)	27 (40.9)	NS
**OHSS**			
**Mild (%)**	43.9	56.1	NS
**Moderate (%)**	5.7	6.1	NS
**Severe (%)**	1.9	1.5	NS

(BMI: body mass index; hCG: human chorionic gonadotropin; MII: meiosis II; D3: third day of embryo development; OHSS: ovarian hyperstimulation syndrome - mild: presence of pain (relieves with scopolamine and/or paracetamol) and/or nausea and/or vomiting; moderate: increase in abdominal volume with evidence of clinical ascites, pain (relieves with codeine), without the need for hospitalization; severe: need for hospitalization due to respiratory distress and/or oliguria).

The number of mature eggs, the clinical pregnancy rate and the frequency of OHSS were similar between the groups. The number of frozen embryos was higher in the Friendly group (Friendly protocol: 7±4.7 *vs*. Conventional protocol: 5.3±2.2, *p*=0.02). The other laboratory variables also showed no significant difference between users of the Friendly or the Conventional protocol (Table 2).

**Table 2.** Multiple linear regression related to the number of mature oocytes (MII).

Variables	Crude Model (IC 95%)	Adjusted Model (CI 95%)	*p*
Friendly *vs*. Conventional	(0.89-1.31)	(0.99-1.61)	0.06
Age (years)	(0.95-0.99)	(0.95-1.02)	0.4
Time of infertility	(0.98-1.05)	(0.96-1.04)	0.9
AFC (basal)	(1.01-1.03)	(1.01-1.03)	0.01
Number of clinic visits	(0.87-1.21)	(0.91-1.29)	0.5
Male factor	(0.08-1.25)	(0.78-1.26)	0.95
Ovulatory factor	(1.05-1.63)	(0.79-1.41)	0.71
Tubal factor	(0.68-1.33)	(0.56-1.18)	0.28
Uterus factor	(0.47-2.11)	(0.64-2.32)	0.55

AFC: antral follicle count.

### Multiple linear regression

The regression model demonstrated that the ovulatory factor, the tubal factor and the number of visits to the clinic were not predictors of the number of mature oocytes. Among the variables analyzed, only AFC was an independent predictor of the number of MII oocytes (*p*<0.01) (Table 2).

## DISCUSSION

The present study demonstrated that the Friendly protocol is as effective as the Conventional protocol, with a similar number of mature oocytes and clinical pregnancy rate. It is also safe for oocyte donors submitted to IVF presenting similar OHSS risk. AFC was the only variable related to number of mature eggs. In addition, the fewer number of visits to the clinic among donors who used the Friendly protocol may be more comfortable (less injections, less loss of working days).

To date, there are no studies that evaluated the simultaneous use of corifollitropin alpha, clomiphene citrate and progestogen in an oocyte donation program. However, the use of long-term gonadotropin associated with progestogen (desogestrel) for LH suppression has been previously evaluated in oocyte donors in Spain. In this study, COS considered the daily use of gonadotropins from the seventh day after using corifollitropin alpha. Similar to our results, the authors found that the number of mature oocytes and the clinical pregnancy rate was similar between users of progestogen (n=29) and the GnRH antagonist (n=25). Although younger, Spanish women had fewer mature oocytes than the participants in the present study. This result may be a consequence of fact that only women with AFC≥20 were included in it. The Spanish authors did not report on the OHSS and early ovulation rate in the retrospective analysis performed by the review of medical records (Martínez *et al*., 2019).

Another retrospective study evaluated the association of corifollitropin alpha with medroxyprogesterone acetate in normal and high responders. In this case series without comparison with other protocols, the authors did not find cases of early ovulation (assessed by the measurement of luteinizing hormone) and there were no patients with OHSS, but the authors did not present the data subdivided according to the ovarian reserve (Huang *et al*., 2018). In the present study, early ovulation (visualization of an image suggestive of the corpus luteum on ultrasound) was not more frequent among users of progestogen for pituitary block, a characteristic already confirmed by other authors in users of medroxyprogesterone acetate with daily gonadotropins, evaluated in randomized trials , both in the comparison with agonist cycles (Wang *et al*., 2016), and in the comparative analysis between the cycles with progestogen and the GnRH antagonist, used for endogenous LH suppression (Beguería *et al*., 2019). The use of progestogen for pituitary block requires embryo freezing, which could represent a disadvantage for delaying treatment completion. However, most high responders need the freeze-all strategy due to the risk of OHSS.

An Australian pilot study compared the use of corifollitropin alpha + clomiphene citrate with the use of gonadotropins in the Conventional protocol (recombinant FSH plus GnRH antagonist) for normal responders, and found that the number of oocytes, the number of frozen embryos and the clinical pregnancy rate was lower among users of long-acting gonadotropin. However, the number of participants [75 women (25 in the citrate clomiphene + corifollitropin alpha group and 50 women in the Conventional protocol group)], the longest infertility time in the corifollitropin alpha group, the administration of long-acting gonadotropin on day 6, the use of additional gonadotropins only after the 13^th^ day of COS and the lower endometrial thickness in the corifollitropin alpha group may have interfered in the results, since the authors did not perform the regression analysis (Zander-Fox *et al*., 2018). According to our results, the association of corifollitropin alpha + clomiphene citrate in continuous regimen during the COS promoted different results in young hyper responders with potential for oocyte donation, in comparison with normal responder women in the Australian study.

The study of corifollitropin alpha in high responders (potential oocyte donors) is limited by the risk of OHSS. However, two studies considered the use of this drug with a GnRH antagonist, trigger with a GnRH agonist and freeze all in high responder women (Hwang *et al*., 2018; Revelli *et al*., 2020). One of the studies was carried out with 40 Taiwanese women with PCOS who received long-term gonadotropin and FSHr. In this case series, the number of MII oocytes was similar to that found in the present study (average 18 oocytes). On the other hand, Taiwanese women did not present moderate or severe OHSS, and the authors considered that this occurred due to the reduced casuistry and the strategies to reduce the risk of OHSS (freeze -ll, trigger with GnRH agonist and use of cabergoline) (Hwang *et al*., 2018). In addition, there was no Control group to compare the results of these authors. In our study, the rates of moderate and severe OHSS were 5.7% and 1.9%, respectively; among users of the Friendly protocol with the same strategies (except for the use of cabergoline) for reducing the OHSS risk (these results were similar to those described in the literature) (Golan *et al*., 1989; Schenker & Ezra, 1994; Delvigne & Rozenberg, 2002).

The other study was a trial that considered the randomization of 43 Italian high responders (AFC>15) for the use of corifollitropin alpha on the second or fourth day of the menstrual cycle (poor and normal responders were also evaluated). In this study, the mean number of MII oocytes obtained was 10 in both groups, a lower number than that seen in our study. However, the average age of the participants in the Italian study was approximately 34 years, while in the present study the average age was 30 years, a characteristic that may justify these different findings. In addition, women with PCOS and BMI > 28 kg/m^2^ were excluded from the Italian study, which could explain possible divergences in the results. The frequency of OHSS was 25.3% with the freeze-all strategy, but the authors did not describe how OHSS was defined (Revelli *et al*., 2020) and, unlike the present study, they also did not consider a group with a Conventional protocol.

In addition to safety (OHSS risk) and efficacy (number of oocytes and clinical pregnancy rate), the Friendly protocol was associated with fewer visits to the clinic. This finding is of great value for the current days when the pandemic by COVID-19 requires social isolation to contain the spread (Lewnard & Lo, 2020). Fewer visits to the clinic decreases the commuting of people, reduces lost days of work, allows better predictability and treatment planning, and can optimize activities in human reproduction clinics. In addition, the reduction in returns during COS and the lower number of injections can increase satisfaction and comfort, and can contribute to reducing IVF costs, but these characteristics still need to be proven.

Despite the same number of MII oocytes obtained from follicular puncture between the groups in the present study, the use of the Friendly protocol was associated with a greater number of frozen embryos. This may had been due to ovulatory factor of infertility and be more frequent in the Conventional protocol group. As anovulation can be associated with worsening oocyte quality and competence (Patel & Carr, 2008), the number of embryos may be lower in these women. In the case series reported in Taiwan, women with PCOS had a higher number of frozen embryos compared to our study, but the authors considered only embryos frozen in D3 (Hwang *et al*., 2018). The number of embryos frozen in the Italian study was not reported (Revelli *et al*., 2020).

To reduce the limitation of the retrospective analysis, we considered all oocyte donors subjected to IVF during the period covered in the present study. Another limitation is the fact that, in the package insert of corifollitropin alpha, the medication should not be used for women with AFC≥20 (off label use), a characteristic that limited the more frequent use of long-acting gonadotropin.

In conclusion, the Friendly protocol seems to be safe and effective in relation to the Conventional protocol for high responder oocyte donors. The fewer number of visits to the clinic with the Friendly protocol can be a great ally in oocyte donation programs. We need randomized controlled trials to evaluate the effectiveness in relation to the live birth rate, the degree of satisfaction and the cost of this alternative for oocyte donation.
